# Sex differences in gait utilization and energy metabolism during terrestrial locomotion in two varieties of chicken (*Gallus gallus domesticus*) selected for different body size

**DOI:** 10.1242/bio.013094

**Published:** 2015-09-24

**Authors:** Kayleigh A. Rose, Robert L. Nudds, Patrick J. Butler, Jonathan R. Codd

**Affiliations:** 1 Faculty of Life Sciences, University of Manchester, Manchester M139PT, UK; 2 School of Biosciences, University of Birmingham, Birmingham B152TT, UK

**Keywords:** Birds, Metabolic rate, Sexual dimorphism, Gravidity, Posture, Mechanics

## Abstract

In leghorn chickens (*Gallus gallus domesticus*) of standard breed (large) and bantam (small) varieties, artificial selection has led to females being permanently gravid and sexual selection has led to male-biased size dimorphism. Using respirometry, videography and morphological measurements, sex and variety differences in metabolic cost of locomotion, gait utilisation and maximum sustainable speed (*U*_max_) were investigated during treadmill locomotion. Males were capable of greater *U*_max_ than females and used a grounded running gait at high speeds, which was only observed in a few bantam females and no standard breed females. Body mass accounted for variation in the incremental increase in metabolic power with speed between the varieties, but not the sexes. For the first time in an avian species, a greater mass-specific incremental cost of locomotion, and minimum measured cost of transport (CoT_min_) were found in males than in females. Furthermore, in both varieties, the female CoT_min_ was lower than predicted from interspecific allometry. Even when compared at equivalent speeds (using Froude number), CoT decreased more rapidly in females than in males. These trends were common to both varieties despite a more upright limb in females than in males in the standard breed, and a lack of dimorphism in posture in the bantam variety. Females may possess compensatory adaptations for metabolic efficiency during gravidity (e.g. in muscle specialization/posture/kinematics). Furthermore, the elevated power at faster speeds in males may be linked to their muscle properties being suited to inter-male aggressive combat.

## INTRODUCTION

Many avian species exhibit sexual dimorphism in morphology, physiology and behaviour, linked to differential specialization of the sexes towards mate competition, reproduction and parental care (Dunn et al., 2001). With the few exceptions of reverse sexual size dimorphism, where females are the larger sex (Reynolds, 1972; Hakkarainen et al., 1996; Pande and Dahanukar, 2012), males are often larger than females and these size differences are more pronounced in cursorial species (Hoglund, 1989). Furthermore, the relative proportions of the skeleton (Baumel, 1953), skeletal muscle and viscera may differ between the sexes (Hammond et al., 2000). Physiological performance traits (e.g. maximum aerobic capacity, maximum speed, endurance and metabolic costs) may also be expected to be sex-specific (Husak and Fox, 2008). Previous studies investigating physiological differences between the sexes in birds have focused on maximum performance and aerobic limits and/or scopes (Chappell et al., 1996, 2011; Hammond et al., 2000). Despite well documented influences of body size and shape on the mechanics and energetics of locomotion (Taylor et al., 1982; Alexander and Jayes, 1983), however, the influence of sexual dimorphism on locomotor performance in birds has been given little attention (Brackenbury and Elsayed, 1985; Lees et al., 2012; Rose et al., 2014).

The metabolic cost of terrestrial locomotion has been investigated across a wide range of avian species. Most studies have focused on interspecific comparisons to understand scaling patterns with respect to body mass (*M*_b_) and deviations from these patterns associated with body form and locomotor specialization. Usually in these studies, only one sex is considered (Nudds et al., 2010); the sex of the experimental animal is not specified (Taylor et al., 1971, 1982; Fedak et al., 1974; Pinshow et al., 1977; Roberts et al., 1998; White et al., 2008), or male and female data are pooled (Bamford and Maloiy, 1980; Bruinzeel et al., 1999; Ellerby et al., 2003; Rubenson et al., 2004; Ellerby and Marsh, 2006; Watson et al., 2011; Tickle et al., 2013).

The potential for sex differences in locomotor performance has been investigated in very few avian species and different studies have produced varying results. For example, male Svalbard rock ptarmigan (*Lagopus muta hyperborea*) were shown to have lower mass-specific metabolic power (*P*_met_; W kg^−1^) requirements than females at any given treadmill speed, despite the sexes sharing similar *M*_b_ (Lees et al., 2012). Furthermore, males achieved greater maximum sustainable speeds (*U*_max_) by 50% and used aerial running gaits, whereas females did not (Lees et al., 2012). These results are consistent with the life history differences between the sexes, whereby male ptarmigan defend vast territories to secure mates and females, who are less active, provide parental care to chicks (Steen and Unander, 1985; Unander and Steen, 1985). In contrast, in the common eider (*Somateria mollissima*), a diving bird, no sex differences in gait choice, *P*_met_ or *U*_max_ were found despite males being 16–18% heavier than females (Rose et al., 2014). The similar locomotor performance of the sexes in eiders is consistent with the short amount of time that each sex spends using terrestrial locomotion, which is important for spring breeding and incubation, but not for securing mates (Portugal and Guillemette, 2011). Without knowledge on the morphological sexual dimorphisms of a species, however, it is difficult to understand any underlying mechanisms behind differences in locomotor performance.

**List of abbreviations**

CoM: centre of mass
CoT_min_: minimum cost of transport
CoT_net_: net cost of transport
CoT_tot_: total cost of transport
*E*_kh_: horizontal kinetic energy
*E*_kv_: vertical kinematic energy
*E*_p_: potential energy
net-*P*_met_: net metabolic power
*P*_met_: metabolic power
RMR: resting metabolic rate
*U*: speed
*U*_max_: maximum sustainable speed
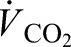
rate of carbon dioxide production
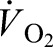
rate of oxygen consumption

Domestic layer chickens (*Gallus gallus domestics*) are a useful species with which to investigate sex constraints on locomotor performance. Not only has artificial selection led to females being permanently gravid but male-biased sexual size dimorphism is common to both wild ancestral and derived chickens due to sexual selection (Remes and Szekely, 2010). The sex-specific behaviours (Guhl et al., 1945; Schutz et al., 2001), morphologies and physiologies (Mitchell et al., 1931; Whitehead, 2004; Remes and Szekely, 2010) of layer breeds are also well documented. For example, males compete with one another for social status, territory and access to females through sustained, aggressive, combats. Furthermore, males partake in courtship activities including feeding, crowing (Chappell et al., 1995; Horn et al., 1995; Wilson et al., 2008), wing dipping and flapping (Chappell et al., 1997). Females, in comparison, invest energy in reproduction (van Kampen, 1976a) and are the sole providers of parental care. To suit these specializations, males possess greater relative anatomical weights of the bones, skeletal muscles, heart and blood, whilst females outweigh males in digestive components, flesh and fat (Mitchell et al., 1931; Hammond et al., 2000).

In a study by Brackenbury and Elsayed (1985), it was hypothesized that the sexes of layer chickens would differ in the metabolic cost of locomotion due to differences in the proportions of total metabolic energy devoted to reproduction (Brackenbury and Elsayed, 1985). Yet, no differences in mass-specific metabolic rates or the incremental cost of locomotion (also known as the minimum cost of transport, CoT_min_: J kg^−1^ m^−1^) were found (Brackenbury and Elsayed, 1985). This lack of a difference is despite the fact that interspecific scaling of the CoT_min_ with *M*_b_, would predict larger males to have a lower CoT_min_ than smaller females. The male and female chickens in (Brackenbury and Elsayed, 1985), however, were from different strains meaning their results are difficult to interpret. Sex differences may not be consistent across chicken strains, which can differ markedly in size and other morphological measurements, depending on the reasons for which they were selectively bred (Paxton et al., 2010).

In this study, we used videography and respirometry to compare male and female gait utilization, *U*_max_ and metabolic rates over a range of treadmill speeds in standard breed (large, L_♂_ and L_♀_) and bantam (miniature, B_♂_ and B_♀_) varieties of leghorn chicken. We tested the hypothesis that sex would lead to greater differences in locomotor energy metabolism than variety, as the varieties are expected to be physiologically and geometrically similar (Rose et al., 2015). In addition, using morphological measurements taken from the birds, we compared the CoT of the birds at equivalent values of dimensionless speed defined by the Froude number (Fr=*U*^2^/*gh*_hip_, where *U* is walking speed, *g* is acceleration due to gravity and *h*_hip_ is hip height) (Alexander and Jayes, 1983). Gravid females were expected to show a lower capacity for locomotion than males through a lower *U*_max_ and fewer gaits utilized.

## RESULTS

### Sexual dimorphism

As expected *M*_b_, *h*_hip_ and Σ *l*_seg_ (the sum of the hind limb skeletal element lengths) were greater in the standard than in the bantam variety (Table 1). *M*_b_ was also 27% and 34% greater in males than in females in the small and large varieties, respectively (Table 1). Similarly, Σ *l*_seg_ was 16% and 20% greater in males than in females in the small and large varieties, respectively (Table 1). Therefore, the sexual size dimorphism of these varieties did not scale geometrically, and was greater in the standard breed. An interaction between variety and sex for Σ *l*_seg_ was found because of a greater difference in size between L_♂_ and B_♂_ (54.67 mm), than between L_♀_ and B_♀_ (38.63 mm). A significant interaction between variety and sex for *h*_hip_ was also found because *h*_hip_ was 33.04 mm taller in B_♂_ compared to B_♀_, whereas in the standard breed, *h*_hip_ was 21.60 mm taller L_♀_ compared to L_♂_ (the opposite pattern) (Table 1). Consequently, sexual dimorphism in limb posture index (*h*_hip_:Σ *l*_seg_) was present in only the standard variety, whereby female limb posture was 23% more erect than that of the males (Table 1).

**Table 1.**
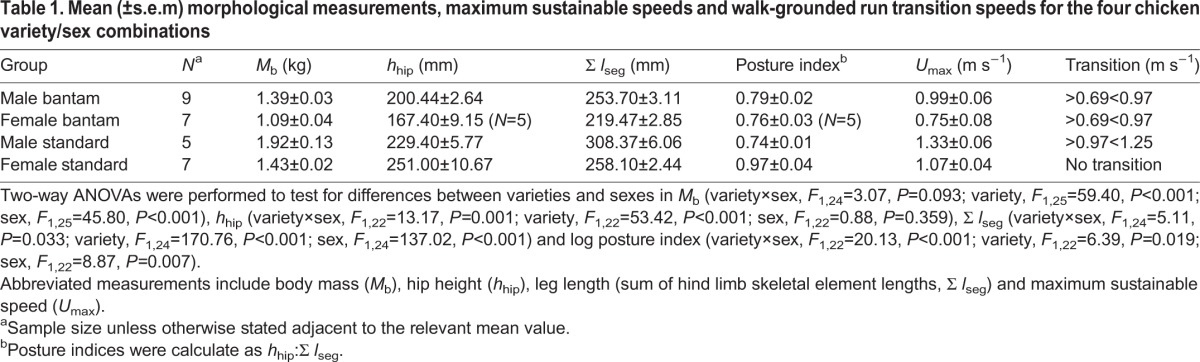
**Mean (±s.e.m) morphological measurements, maximum sustainable speeds and walk-grounded run transition speeds for the four chicken variety/sex combinations**

### Gaits

**Fig. 1.**
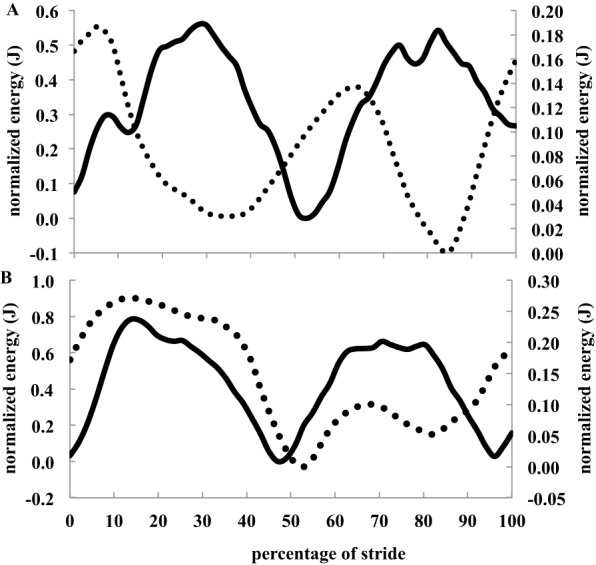
**Examples of typical mechanical energy fluctuations of the CoM.** (A) Walking gait (0.69 m s^−1^ in a L_♂_, 2.19 kg). (B) Grounded running gait (1.39 m s^−1^ in a L_♂_, 2.19 kg). Solid lines and the left *y*-axis represent horizontal kinetic energy (*E*_kh_) of the CoM, and the dotted lines and the right *y*-axis represent vertical kinetic plus potential energies (*E*_kv_+*E*_p_).

With exception of L_♀_ and B_♂_, which shared similar *U*_max_ (Table 1), *U*_max_ differed between groups (*Χ*^2^=17.41, d.f.=3, *P*<0.001) and was greater in males compared to females by 15% and 25% in bantam and standard breed leghorns, respectively. None of the birds in this study had duty factors below 0.5; therefore, they did not use aerial running gaits. In L_♂_, the maximum speed (*U*) at which the horizontal kinetic energy (*E*_kh_) of the body centre of mass (CoM) was observed to fluctuate out-of-phase with the sum of the vertical kinetic and potential energy (*E*_kv_+*E*_p_) of the CoM (walking gait mechanics, Fig. 1A) was 1.11 m s^−1^ (2 of 5 individuals). From 1.11–1.39 m s^−1^ the *E*_kh_ and *E*_kv_+*E*_p_ of their CoM were in-phase (Fig. 1B), indicating that they used grounded running gaits. At the *U*_max_ of the L_♀_, however, the *E*_kh_ and *E*_kv_+*E*_p_ of the CoM were out-of-phase indicating that they were still walking. In bantams of either sex, *E*_kh_ and *E*_kv_+*E*_p_ of the CoM were out-of-phase at speeds up to and including 0.83 m s^−1^, and in-phase from speeds of 0.83 m s^−1^ and greater, indicating that the sexes utilized walking and grounded running gait mechanics over similar speed ranges. However, only 3 of 7 females could sustain 0.83 m s^−1^, at which speed one individual was still walking. The same 3 B_♀_ could sustain 0.97 m s^−1^ and were all grounded running at this speed. Therefore, most B_♀_ and all L_♀_ were either unwilling or incapable of performing a grounded running gait.

### Resting metabolic rates

During quiet standing, RMR (*P*_met_, W) was positively correlated with *M*_b_ (Table 2) and the slopes and intercepts of this relationship were similar between sexes and varieties (means were B_♂_: 10.70±0.50, B_♀_: 8.54±0.41, L_♂_: 13.80±0.66 and L_♀_: 9.25±0.44). Likewise, mass-specific RMR (*P*_met_, W kg^−1^) was similar (Table 2) between sexes and varieties (means were B_♂_: 7.85±0.27, B_♀_: 7.13±0.57, L_♂_: 7.21±0.48 and L_♀_: 7.24±0.42).

**Table 2.**
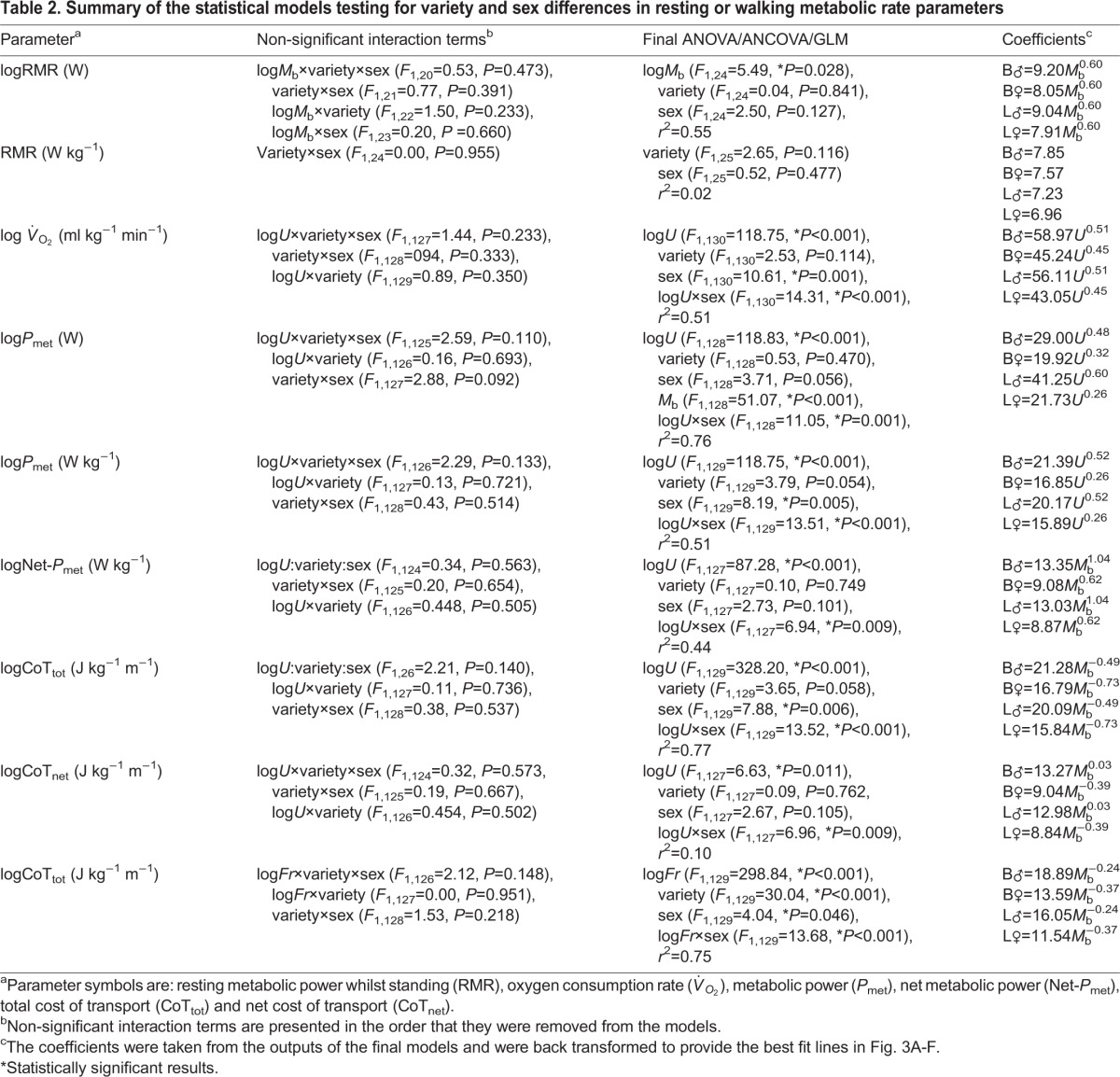
**Summary of the statistical models testing for variety and sex differences in resting or walking metabolic rate parameters**

### Walking metabolic power

**Fig. 2.**
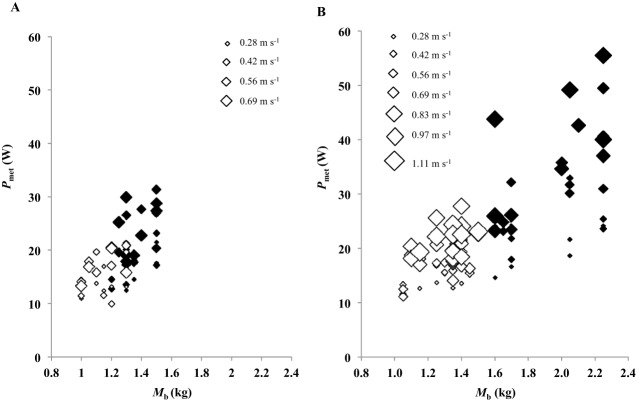
**Metabolic power versus body mass during walking gait.** Black and white symbols represent males and females, respectively, in bantam leghorns (A) and standard breed leghorns (B). The size of the diamond represents the magnitude of the speed.

**Fig. 3.**
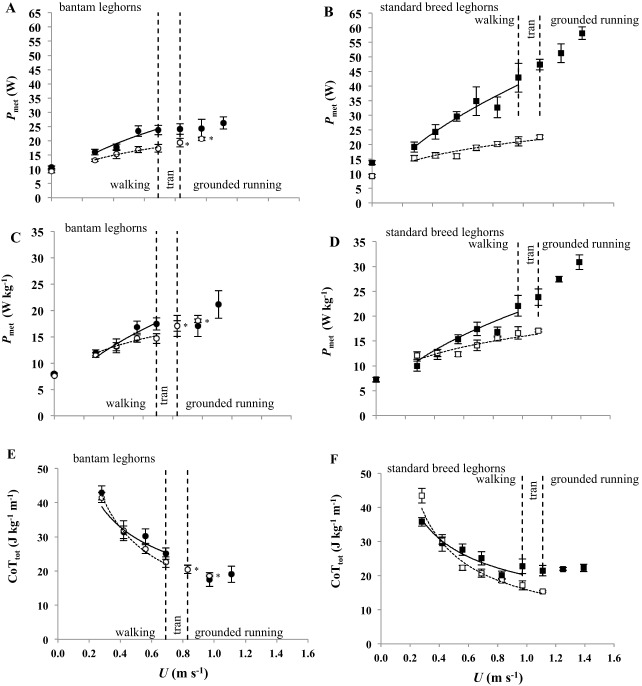
**Metabolic parameters as a function of treadmill speed.** Black and white symbols represent males and females, respectively. Circles and squares represent bantam and standard breed leghorns, respectively. (A-B) Metabolic power (*P*_met_, W)*.* (C-D) Mass-specific metabolic power. (E-F) Total mass-specific cost of transport (CoT_tot_, J kg^−1^ m^−1^). Dashed vertical lines represent the greatest walking speed and the lowest grounded running speed. L_♀_ used walking mechanics across their full speed range. Asterisks indicate where B_♀_ sample size was *N*=3. Equations for the lines of best fit (males=solid lines and females=dashed lines) are given in Table 2. Data is represented as mean (±s.e.m).

Absolute *P*_met_ (W) was correlated with *M*_b_ and *U* during walking (Fig. 2A-B) and increased curvilinearly (Fig. 3A-B) with *U* in all birds (Table 2). The incremental response to *U* was steeper in the bantams compared to the standards, but this difference was not significant when accounting for *M*_b_ (Table 2). *M*_b_, however, did not explain the greater incremental response to *U* in males than in females (Table 2).

Mass-specific *P*_met_ (W kg^−1^) was positively correlated with *U* in all bird groups (Fig. 3C-D) and was best described by power curves. The exponents of these curves were common to both varieties with the incremental increase in mass-specific *P*_met_ with *U* greater in males compared to females (Table 2). Mass-specific *P*_met_ was lower across all *U* in the males of the larger variety than in males of the bantams, and likewise in females.

Calculating mass-specific net-*P*_met_, by subtracting *P*_met_ during quiet standing from *P*_met_, did not account for this sex difference (Table 2), but did reduce the net metabolic rates (intercepts) of the bantam variety relative to the large variety (Table 2). Again, net mass-specific *P*_met_ increased with *U*, with higher exponents and intercepts in males than in females, and similar exponents, and intercepts for the males and females of each variety (Table 2). Therefore, the sexes shared similar metabolic rates at low speeds (Table 2); however, with increasing *U*, metabolic rates increased at a faster rate in males compared to females, indicating that to move at faster speeds is more costly to males than to females.

As has been found previously in exercising domestic chickens (Brackenbury and Elsayed, 1985), respiratory exchange ratios (RERs) were close to 1 across all treadmill speeds (B_♂_,: 1.09 [1.06-1.12], B_♀_: 1.10 [1.08-1.17], L_♂_,: 1.09 [1.04-1.20] and L_♀_: 1.14 [1.08-1.21], means and [ranges]). RER increased positively with *U*, which may suggest a greater anaerobic contribution to metabolism with increasing *U*. No signs of fatigue (trouble maintaining balance, head or wing droopiness) or post exercise oxygen deficit on the gas traces were found however, to suggest a large amount anaerobic respiration by the muscles. Statistical analyses on mass-specific 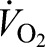 with speed produced the same statistical outcomes as mass-specific *P*_met_ (Table 2).

### Walking cost of transport

**Fig. 4.**
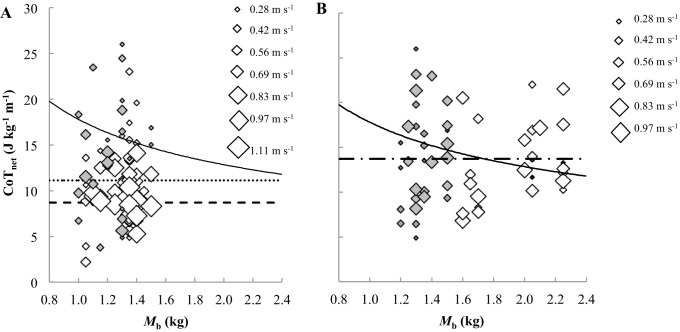
**Net cost of transport versus body mass across the range of walking speeds and mean minimum measured costs of transport.** Grey and white diamonds represent bantam and standard breed leghorns, respectively. The size of the diamond represents the magnitude of the speed. Solid curves are Rubenson et al's (2007) interspecific allometric relationship between walking CoT_min_ and *M*_b._ Mean CoT_min_ is represented by a dotted line for B_♀_, a dashed line for L_♀_ and a dotted and dashed line for all males. (A) Female CoT_net_ decreased as a function of speed and the majority of their values were below the predicted CoT_min_. (B) Male CoT_net_ was independent of speed and both varieties shared the same mean CoT_min_ closer to the prediction for the standard breed variety.

The total metabolic cost of transport (CoT_tot_, J kg^−1^ m^−1^) decreased curvilinearly with *U* in both varieties and sexes (Fig. 3E & F). The rate of decrease in CoT_tot_ was similar between varieties; however, the intercepts were lower in the larger variety compared to the bantams by ∼1 J kg^−1^ m^−1^ (Table 2). The incremental decrease in CoT_tot_ with *U* was greater in females than in males (Table 2). The change in mass-specific net metabolic cost of transport (CoT_net_, J kg^−1^ m^−1^) with *U* (Fig. 4) was almost independent of speed (small positive increase) in males, but decreased curvilinearly in females (Table 2). Consequently, the minimum measured CoT_net_ in females occurred at their maximum walking speed and was 11.79 and 8.67 J kg^−1^ m^−1^ in B_♀_ and L_♀_, respectively (Fig. 4A). These values are lower than predictions (B_♀_=17.09 and L_♀_=15.40 J kg^−1^ m^−1^) based on interspecific allometry [CoT_min_=17.80*M*_b_^−0.47^ (Rubenson et al., 2007)] of the minimum measured CoT_net_ for walking gaits (Fig. 4A). The CoT_net_ of the females was lower than the CoT_min_ predicted by interspecific allometry across the majority of their speed range, excluding the two slowest speeds (0.28 and 0.42 m s^−1^) (Fig. 4A). The CoT_net_ values of the males were scattered either side of the CoT_min_ prediction, uncorrelated with *U* and not significantly different between varieties (Fig. 4B).

### Froude corrections

**Fig. 5.**
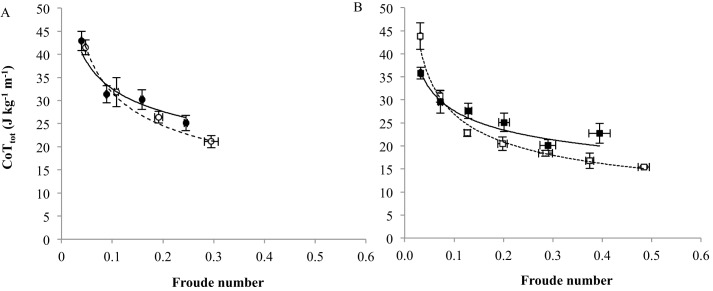
**Total cost of transport versus Froude number.** Bantam leghorn data is shown as circles. (A) and standard breed leghorns data as squares (B). Black and white symbols represent males and females, respectively. Data is represented as mean (±s.e.m).

The sex differences in CoT_tot_ at a given *U* may exist because the locomotion of the sexes is not dynamically similar. When calculated using weight (N) instead of *M*_b_, the CoT_tot_ reduces to a dimensionless parameter (Fish et al., 2000). The dynamic similarity hypothesis poses that geometrically similar animals moving with equal ratios of gravitational and inertial forces acting on their body CoM (i.e. at equal Fr) will incur a similar CoT (Alexander and Jayes, 1983). CoT_min_ decreased curvilinearly with Fr at a faster rate in female than in male leghorns (Fig. 5A-B).

The maximum Fr recorded, at which the females were still walking and incurred their CoT_min_ was greater than that for males. At the Fr equivalent to the *U*_max_ of the males, the CoT was already lower in females than in males. Female leghorns, therefore, carry a unit of their *M*_b_ over a unit of distance with greater economy of energy use than males.

### Grounded running in males

During grounded running gaits in the males, mass-specific *P*_met_ (W kg^−1^), was ∼5.75 W kg^−1^ greater in the L_♂_, compared to B_♂_ across all *U* (Table 3; Fig. 3C-D). Calculating net mass-specific *P*_met_ (W kg^−1^) increased this difference between varieties to 9.18 W kg^−1^ (Table 3). Since *P*_met_ during quiet standing was the same between varieties, the reduction in grounded running *P*_met_ in the standard breed relative to the B_♂_ upon calculating net-*P*_met_ may indicate change in the postural cost of locomotion during a grounded running gait. CoT_tot_ during grounded running was 7.76 J kg^−1^ m^−1^ greater in L_♂_, than in B_♂_. Similarly, CoT_net_ was 6.27 J kg^−1^ m^−1^ greater in the standard variety. Neither *P*_met_, net mass-specific *P*_met_, CoT_tot_ nor CoT_net_ changed with *U* in either variety (Table 3). When compared to interspecific allometric predictions of running using CoT_min,_=12.91*M*_b_^−0.346^ (Rubenson et al., 2007), the measured B_♂_ value is similar (B_♂_ measured, predicted: 9.63 and 10.30 J kg^−1^ m^−1^), but the measured L_♂_, value is greater (large measured predicted: 15.90 and 11.52 J kg^−1^ m^−1^). Therefore, during a grounded running gait, L_♂_, have a poorer economy of energy use than do B_♂_.

**Table 3.**
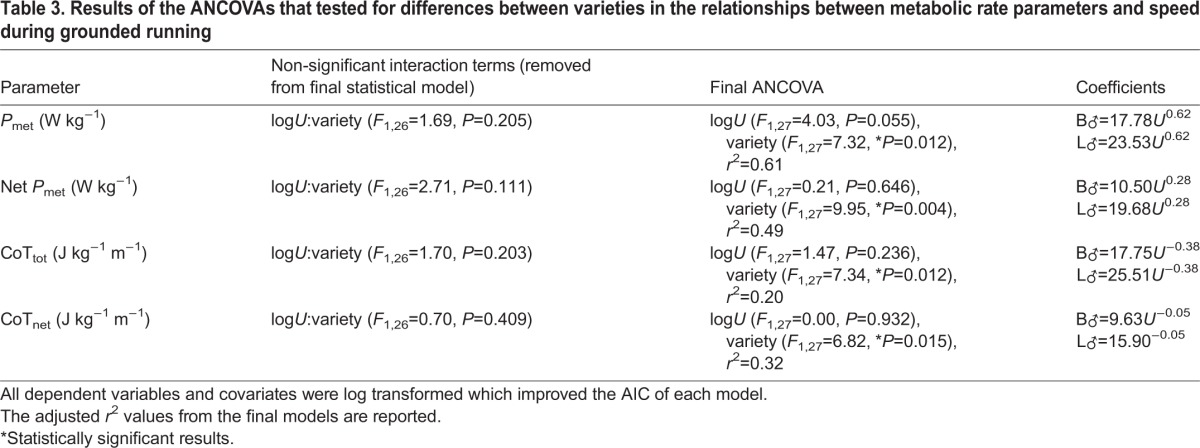
**Results of the ANCOVAs that tested for differences between varieties in the relationships between metabolic rate parameters and speed during grounded running**

## DISCUSSION

The principal aim of this study was to determine the influence of sex on locomotor performance in standard breed (large) and bantam (small) leghorn chickens. Differences in the incremental increase in walking *P*_met_ with *U* between the varieties were negated by mass-correction, but mass-correction did not remove the observed sex differences. In both varieties, *P*_met_ increased more rapidly with walking *U* in males than in females, indicating that to walk at faster speed was more costly in males, relative to females. This is the first evidence of a greater CoT_min_ in a male bird when compared to a female. Our study is also the first to compare the CoT of the sexes over a similar range of Froude numbers in a species of bird. After negating the effects of body size and speed, the sex differences in CoT_tot_ were shared by the two varieties, despite them exhibiting dissimilar sexual dimorphism in limb posture. While L_♀_ were 23% more upright than L_♂_, no sex difference in posture was present in the bantam variety.

In both varieties, females were lighter than males and had a lower CoT_min_, which contrasts to the expected negative allometry of CoT_min_ with increasing *M*_b_ (see solid line in Fig. 4A,B) across species (Taylor et al., 1982; Rubenson et al., 2007). It is widely accepted, however, that there is no independent effect of *M*_b_ on CoT_min_ (Pontzer, 2007). Furthermore, a growing body of evidence supports the hypothesis that the interspecific increase in limb erectness with *M*_b_ is linked to the allometry of CoT_min_ (Mcmahon et al., 1987; Griffin et al., 2004; Pontzer, 2007; Reilly et al., 2007; Nudds et al., 2009; Rose et al., 2015). At the intraspecific level, however, limb posture is not expected to change with *M*_b_ (Griffin et al., 2004; Day and Jayne, 2007; Rose et al., 2015). Another reason why the measured sex differences in CoT_min_ were unexpected is that females leghorns have lower ratios of skeletal muscle mass: visceral and reproductive mass, relative to males (Mitchell et al., 1931). Since the muscle force required to support body weight is considered the principal contributor to the metabolic cost of terrestrial locomotion (Taylor et al., 1980), above other costs such as swinging the limb (Marsh et al., 2004), and maintaining posture (Weyand et al., 2009), the females might be expected to incur a greater metabolic cost of locomotion per unit *M*_b_. Adding loads to the backs of mammals to manipulate *M*_b_, for example, leads to an increase in net locomotor metabolic rate, greater in proportion than the proportional increase in mass (McGowan et al., 2006). In the few avian species examined to date, however, an extra gram of back load was carried at a cost equal to (Tickle et al., 2010), or less than (Marsh et al., 2006; McGowan et al., 2006; Tickle et al., 2013) carrying a gram of original *M*_b_. If the hens carry each gram of reproductive load at a cost less than carrying each gram of the remaining *M*_b_, this could lead to the observed lower than expected CoT after dividing by total *M_b_*. Similar, to a previous finding in laying hens (van Kampen, 1976b), the CoT_min_ of the females in this study was lower than that predicted using interspecific allometry. We expect, however, that more than just the exceptional load carrying ability of some birds compared to mammals is responsible for the low female CoT relative to *M*_b_ and relative to male CoT.

Sexual dimorphism in physiological performance is often associated with sex-specific adaptations that have resulted from the differential selective pressures on the sexes given their different life histories (Rogowitz and Chappell, 2000; Shillington and Peterson, 2002; Husak and Fox, 2008; Lees et al., 2012). Female chickens invest metabolic energy in gravidity (van Kampen, 1976a; Gloutney et al., 1996). Selection may be expected to act on the female's ability to carry eggs with metabolic economy of force generation. The evolution of compensatory traits that alleviate the potential costs of exaggerated sexually selected morphologies is usually considered from the male perspective (Husak and Swallow, 2011). Gravid female lizards have been shown to experience this type of selection (Shine et al., 1998). It is, however, unknown if this occurs in birds. One potential compensatory mechanism in females could be muscular adaptations that promote economical force generation (e.g. shorter fascicle lengths, or an increase in the proportion of slow oxidative muscle fibres). Furthermore, females may employ different gait kinematics (e.g. increased time of foot-ground contact), which allow the recruitment of slower muscle fibres (Kram and Taylor, 1990).

Male chickens, by contrast, invest more energy in terrestrial locomotion than females through maintaining territory, inter-male aggressive behaviour and intersexual courtship activity. Although the influence of these behaviours on daily energy budget is not known, it is interesting to consider why selection has not reduced the metabolic requirements of locomotion in leghorn males, relative to the less active females, as was found in another galliform species (Lees et al., 2012). Perhaps a stronger selection pressure on fighting ability promotes muscle architecture for fast, powerful, and sustained combats that are costly to use at intermediate to high walking speeds. Faster contracting, relatively longer muscle fascicles, and muscles with a greater capacity for force generation might be expected to have elevated power demands. There is precedence for this type of adaptation in birds as sex differences in flight muscle specialization have previously been identified in species where the males partake in fast volant courtship displays and females use high powered locomotion to a lesser degree than the males (Schultz et al., 2001).

As expected, males achieved greater *U*_max_ than females in common with many vertebrate species (Bhambhani and Singh, 1985; Brackenbury and Elsayed, 1985; Shine and Shetty, 2001; Finkler et al., 2003; Lees et al., 2012). Of course, the size difference between the sexes could explain this finding. However, a greater *U*_mas_ in males compared to females is also common to species lacking sexual size dimorphism, but where the males have higher activity levels than females during the mating season (Lees et al., 2012). The greater *U*_max_ in males is likely supported by their specializations for inter-male combat, including relatively larger skeletal muscles, hearts and lungs compared to females (Mitchell et al., 1931). At the same time, a reduction in *U*_max_ and sprint speed in vertebrate females is often associated with the encumbrance of pregnancy or gravidity (Olsson et al., 2000; Shine, 2003; Knight, 2011). One benefit of a lower *U* is that it allows a longer stance phase during which sufficient force can be generated to support body weight. We suspect that the ability to generate sufficient force may limit female *U*_max_, relative to the males, given their lower muscle mass: visceral/reproductive mass ratio.

Females of the two varieties were reluctant or unable to transition to grounded running gait mechanics. It is possible that they avoided higher *U* and grounded running gaits in order to reduce peak forces on their bones and avoid fracture as their bones may be weakened by the provision of medullary calcium towards eggshell formation (Bloom et al., 1941; Whitehead, 2004). This may be particularly pertinent in white leghorns, which are prone to osteoporosis during eggshell construction (Dacke et al., 1993).

## CONCLUSIONS

The sexes of both standard breed and bantam varieties of leghorn chicken differed in all measured aspects of terrestrial locomotion. Males attained greater *U*_max_ compared to females and used a grounded running gait at faster speeds, while gravid bantam females were reluctant to and standard breed females did not. These findings are consistent with the general consensus that gravidity and lower ratios of skeletal muscle:visceral mass in females, constrain locomotion. Our findings are likely the result of a combination of sex-specific adaptations and associated constraints that have resulted from differential selection pressures on the sexes. Furthermore, we suggest that gravid females may possess adaptations for greater metabolic economy of locomotion (e.g. in muscle specialization/posture/kinematics).

## MATERIALS AND METHODS

### Animals

We acquired sexually mature (>16 weeks<1 year old) standard breed (5 male, 1.92±0.13 kg; 7 female, 1.43±0.02 kg, mean±s.e.m.) and bantam (9 male, 1.39±0.03 kg; 7 female, 1.09±0.04 kg, mean±s.e.m.) Leghorn chickens from local suppliers between March and May (breeding season) and housed them in the University of Manchester's Animal Unit. Hens were egg laying and males exhibited secondary sexual morphological characteristics, crowing and aggressive behaviour. Sexes and varieties were housed separately with *ad libitum* access to food (Specialist Poultry Breeder, Small Holder Range, Norfolk, UK: oils and fats: 6%; protein: 18%; fibre: 4.5%; Ash: 12.0%; calcium 4%) and water. Light-dark cycles were fixed at 13:11 h and temperatures at 18–22°C. The birds were trained daily for one week to exercise on a treadmill (Tunturi T60, Turku, Finland), within a Perspex^®^ respirometry chamber. None of the birds was fasted prior to respirometry measurements. The male birds in this study were previously used in (Rose et al., 2015). A UK Home Office Project License held by Dr Codd (40/3549) covered all experimental procedures, which were undertaken with the ethical approval of the University of Manchester Ethics Committee.

### Respirometry

Rates of O_2_ consumption (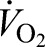, ml min^−1^) and CO_2_ production (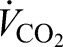, ml min^−1^) were measured from resting (standing) and exercising birds using a flow-through respirometry system (all equipment Sable Systems International^®^, Las Vegas, NV, USA). Different sized chambers were built for large (97.5×53.5×48 cm) and bantam leghorns (66×46.5×48 cm) and a Flowkit 500 pulled ambient air through them at flow rates of 150 and 250 litres min^−1^ respectively. The Flowkit directed a sub-sample (0.11 litres min^−1^) from the main flow through the gas analysis system. Water vapour pressure (WVP) was measured by an RH300 before H_2_O was scrubbed from the sample, using calcium chloride (2–6 mm granular, Merck, Darmstadt, Germany) and passed on to a CA-10A CO_2_ analyser for CO_2_ measurements. Dry air was scrubbed of CO_2_ with a column of soda lime (2–5 mm granular, Sigma Aldrich, Steinheim, Germany) before passed on to an Oxzilla II O_2_ analyser for O_2_ and barometric pressure (BP) measurements. A pump (SS-3) sampled ambient air through a second channel at 0.11 litres min^−1^ and the sample was scrubbed of H_2_O and CO_2_ (as previously described) before being passed through the Oxzilla. The accuracy of the set up (±5% across all treadmill speeds) was validated using a N_2_ injection test (Fedak et al., 1981).

Differential O_2_ concentration (ΔO_2_, ambient O_2_−box O_2_ concentrations) was used in all calculations. CO_2_ traces were base-lined in the absence of a bird, which allowed the calculation of differential CO_2_ (ΔCO_2_). Primary flow rates (*F*) were converted to corrected flow rates (*F*_c_) to account for the H_2_O removed from the samples using Eqn 8.6 from Lighton (2008):
(1)
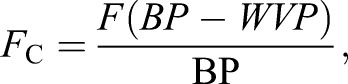

where WVP is water vapour pressure. 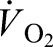 and 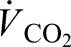 were calculated using Eqns 10.1 and 10.8 from Lighton (2008), respectively:
(2)
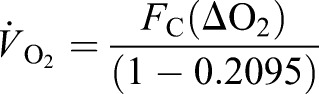

(3)
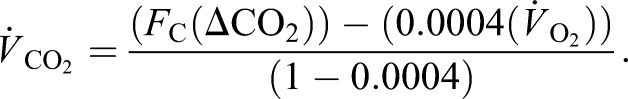



RERs (
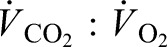
) and their thermal equivalents (taken from Table 12.1 of Brody, 1945) were used to convert 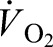 into *P*_met_ (W). To account for potential sex differences in body maintenance and postural metabolic requirements, net-*P*_met_ (locomotor *P*_met_ – resting *P*_met_ during quiet standing) was calculated using values taken from the same trial for each individual bird.

### Trials

Experimental temperatures ranged from 17.5–22.8°C (19.8±1.5°C, mean±s.e.m.). In a single trial, birds were exercised at a maximum of three randomly selected speeds and were given resting intervals of at least 5 min between each period of exercise to recover. The birds were walked at a minimum speed of 0.28 m s^−1^ and at increments of 0.14 m s^−1^ up to the maximum that they could sustain for steady 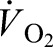 readings (>3 min). The final 1 min of the plateau was used for data analysis. All resting metabolic rates were taken from the final rest period of a trial and birds were given a day of rest between trials.

### Determining gait

The gait mechanics of each bird was determined from video recordings (100 frames s^−1^; HDR-XR520VE, SONY, Japan) taken perpendicular to the direction of travel of the birds (from the left) in all trials. Using Tracker software v2.51 (Open Source Physics) a marked site over the left hip (the CoM) was tracked (min 3 strides) in every film frame to determine the mechanical energy fluctuations using temporal and spatial data. A calibration stick was positioned along the line of travel of a bird passing through digit 3 to avoid any error in measured dimensions that might have arisen due to a bird's displacement from it. The phasing of the CoM fluctuations in horizontal kinetic energy (*E*_kh_) with the sum of its vertical kinetic and gravitational potential energies (*E*_kv_+*E*_p_) was used to determine gait. An out of phase relationship is characteristic of walking gaits and an in-phase relationship of running gaits.

### Statistical analyses

Statistical analyses were performed using the car package version 2.0-12 (Fox and Weisberg, 2011) R 2.14.0 GUI 1.42 Leopard build 64-bit (R Development Core Team, 2011). Morphological measurements were tested for the main effects of sex and variety as well as potential interaction effects using two-way ANOVAS. Resting *P*_met_ and RERs were investigated for sex and variety differences using ANCOVA. *M*_b_ was included in the models as a covariate to compensate for the effects of *M*_b_ and variety and sex were included as fixed factors. The relationships between exercising metabolic rates and *U* were investigated for differences (in slopes and intercepts) between varieties and sexes (both factors) using linear models. Speed was included as the main covariate in each model. For non-mass-specific metabolic parameters, *M*_b_ was included in the models as an additional covariate. For mass-specific metabolic rates, *M*_b_ was not included in the models. All potential interaction terms were considered in the primary models before a step-wise backward deletion of non-significant interaction terms was conducted. For all parameters, the quality of our linear models according to the Akaike's information criterion was improved by log transforming the data. Shapiro–Wilk tests were performed on the standardised residuals generated by each statistical model to ensure that the data conformed to a normal distribution. In the case of the *U*_max_ comparison between groups, the residuals did not conform to a normal distribution even after transformation, so a Kruskal–Wallis test with a Dunn post-hoc test was used. The adjusted *r*^2^ values of the models are reported and unless otherwise stated the means are reported as ±s.e.m.

The influence of speed on metabolic rate is gait dependent in some avian species (Rubenson et al., 2004, 2007; Nudds et al., 2011). Statistical analyses were, therefore, conducted on metabolic data from walking and grounded running gaits, separately. Sex comparisons were conducted for walking gaits only, since very little grounded running data were collected from the females.
